# One drug to treat many diseases: unlocking the economic trap of rare diseases

**DOI:** 10.1007/s11011-020-00617-z

**Published:** 2020-09-14

**Authors:** Karolina Pierzynowska, Teresa Kamińska, Grzegorz Węgrzyn

**Affiliations:** 1 Department of Molecular Biology, Faculty of Biology, University of Gdańsk, Wita Stwosza 59, 80-308 Gdańsk, Poland; 2 Department of Microeconomy, Faculty of Economy, University of Gdańsk, Armii Krajowej 119/121, 81-824 Sopot, Poland

**Keywords:** Rare diseases, Neurodegenerative diseases, Orphan drugs, Economic trap, Hypothesis of one drug for many diseases

## Abstract

There are two major problems with the development of therapies for rare diseases. First, among over 7000 such diseases, the vast majority are caused by genetic defects and/or include neurodegeneration, making them very difficult to treat. Second, drugs for rare diseases, so-called orphan drugs, are extremely expensive, as only a small number of patients are interested in purchasing them. This results in the appearance of a specific economic trap of rare diseases; namely, despite high biomedical, pharmaceutical and technological potential, the development of new orphan drugs is blocked by the economic reality. The purpose of this work was to find a potential solution that might resolve this economic trap of rare diseases. A literature review was conducted, and a hypothesis was formulated assuming that the use of one drug for the treatment of many rare diseases might overcome the economic trap. We provide examples showing that finding such drugs is possible. Thus, a possible solution for the problem of developing orphan drugs is presented. Further preclinical and clinical studies, although neither easy nor inexpensive, should verify whether the hypothesis regarding the possibility of unlocking the economic trap of rare diseases is valid.

## Introduction

There are approximately 7000 rare diseases, and the vast majority of them are particularly difficult to treat (Nguengang Wakap et al. 2020). Among rare diseases, genetic and neurodegenerative disorders are predominant. There are two problems with finding effective drugs for such diseases. First, genetic defects occur in all cells (or their progenitors) of patients; this problem is enhanced by the fact that the majority of these diseases are severe, with devastating symptoms that might be irreversible if the treatment is not started early (Zschocke 2008). In fact, their molecular mechanisms are usually complicated, and even in the case of monogenic disorders, pathomechanisms involve disturbances of many processes occurring at the cellular and organismal levels (Gaffke et al. 2020). However, currently available knowledge and technology allow for discoveries of various treatments that can be potentially effective in rare diseases (Ahmed et al. 2019). On the other hand, the second problem appears to be the major inhibitor of this process. Namely, since the number of patients suffering from one rare disease is very low (by definitions used in the EU and US, a disease is classified rare if it affects fewer than 1 per 2000 people or fewer than 200,000 people, respectively), there is a specific economic problem in the development of drugs for such diseases, called orphan drugs. This is because the cost of developing an orphan drug is as high as that of any other drug (devoted to patients suffering from common diseases) while the number of potential buyers is very low (Jayasundara et al. 2019). Therefore, prices of already approved orphan drugs are extremely high, at the level of hundreds thousands or even millions of dollars/euros (Luzzatto et al. 2018). This causes serious problems for both patients (as the availability of orphan drugs depends on the possibility of obtaining reimbursement) and pharmaceutical companies (as the development of orphan drugs is economically very risky). Because of high costs, insurance companies or governmental agencies (depending on the health insurance system in a particular country) are reluctant to reimburse orphan drugs, making the risk for pharmaceutical companies even higher (Zamora et al. 2019).

In such a situation, when a potential customer is not able to buy the product, a potential payer likely rejects the request for reimbursement; thus, the producer cannot sell the product, and no party can obtain a profit from the drug, even if it can save the lives of patients. Therefore, only relatively few companies are interested in working on novel orphan drugs, despite various benefits established by drug-administrating agencies, including fast tract of drug registration, 7 year exclusivity, 50% tax credits for research and development, and special grants (Luzzatto et al. 2018). Moreover, even if a drug is registered, the risk is still extremely high. The best example is cancellation of the first gene therapy product, Glybera, registered in the EU in 2012. The cost of treatment was over 1 million euros, and during the five years of registration, only one patient was treated. Therefore, the company gave up, the registration has not been prolonged, and Glybera is no longer available to patients (Senior 2017). Intriguingly, another gene therapy product, Zolgensma (onasemnogene abeparvovec-xioi), desired for treatment of infantile spinal muscular atrophy (Pattali et al. 2019), has been registered on May 24, 2019 in USA (Al-Zaidy and Mendell 2019), and then in EU and Japan (Stevens et al. 2020). The therapy was demonstrated to cause improvement in neuro-muscular functions of treated children (Hoy 2019), however, Zolgensma is not able to provide a complete cure (Chen 2020). Nevertheless, the price of the single dose therapy is over 2 million US dollars, which raises concerns about the cost and availability of the drug (Stevens et al. 2020).

Difficulties in availability of expensive therapies can be exemplified by enzyme replacement therapy, registered for treatment of various lysosomal storage diseases (Concolino et al. 2018). This therapy is based on intravenous administration of recombinant enzyme which should replace the function of native enzyme whose activity is otherwise absent or drastically decreased in patient’s cells. The enzyme is provided every week or every two weeks, and the therapy is expected to be lifelong (Lachmann 2020). The economical problems related to high cost of enzyme replacement therapy (several hundred thousand dollars/euros per year; the exact price depends on patient’s body weight as the enzyme dose is given per kg), including problems with reimbursement of the costs, have been recognized earlier (Schlander and Beck 2009). In fact, interruption of this kind of therapy because of refusal of covering costs by insurance companies or governmental health agencies became a real problem in many countries (Solano et al. 2020). Precise economic analyses were performed for enzyme replacement therapy in a couple of diseases. It was calculated that the total average cost of lifetime therapy of Gaucher disease is 5.7 million euros, contrary to estimated costs of the management without specific therapy at the level of 170 thousand euros (van Dussen et al. 2014). Therefore, if a patients is treated, the extra cost of “year free of end organ damage” (YFEOD) is over 400 thousand euros, and “quality-adjusted life year” (QALY) is almost 900 thousand euros (van Dussen et al. 2014). The latter parameter (QALY) was also calculated for enzyme replacement therapy of Pompe disease, giving the amount of over 1 million euros (Schoser et al. 2019). These numbers underline the level of the problem and the dilemma arising when one should decide whether to reimburse the high cost of the therapy or to reject the option of financing a drug which can otherwise save or prolong the patient’s life (or at least significantly improve the quality of life). These difficulties may escalate if more than a few extremely expensive drugs (each for a different disease) are potentially available and one would have to choose which patient can be treated and which must be devoid of the drug. Definitely, health insurance systems are not prepared for financing many extremely expensive therapies which may further discourage pharmaceutical companies to invest in development of orphan drugs in near future.

### The economic trap of rare diseases

The problems described above result in the appearance of the specific economic trap of rare diseases. Despite high biomedical, pharmaceutical and technological potential, the development of new orphan drugs is blocked by economic reality. The fact that the mechanisms of many rare diseases are complex and perhaps require combinations of various treatments or have at least more than one target to achieve expected clinical effects even worsens this situation (Gabig-Cimińska et al. 2015). Therefore, one may ask what can be suggested to escape from this trap? If no solution is proposed, patients suffering from thousands of rare diseases may be left without therapies despite relatively high levels of biomedical knowledge and technological development that might otherwise help millions of people.

### The ‘one drug for many diseases’ hypothesis

We propose the following hypothesis. Since each rare disease concerns a small number of patients and each disease requires a specific drug, the number of potential customers interested in purchasing this drug is low. However, we assume that this problem might be overcome if one drug is effective in the treatment of many diseases. Let us consider a putative drug suitable for a therapy that would cure patients suffering from 10 or even 100 different rare diseases. Two economic factors may emerge that favor the situation, i.e., increase in both market demand (increasing number of patients) and market supply (lower average fixed costs arising for manufacturers). Afterwards, the reimbursement attitude regardless of insurance or governmental agencies would be more fostering and less risky. Then, the number of patients interested in purchasing such a drug would increase dramatically, achieving the level of common diseases, for which the production of drugs is undoubtedly profitable to pharmaceutical companies. This might open a new era in orphan drugs that, in fact, would become nonorphan.

### Theoretical evaluation of the hypothesis

There are some conditions to follow such a scenario. First, researchers and pharmaceutical companies might have to change the thought process from the traditional “one-target” scheme (Carroll 2005; Ramsay et al. 2018). It is commonly accepted now that a “good” drug should have a single biological entity as a target. However, the use of molecules that have various biological activities and different targets might be potentially more useful in the treatment of various diseases. In fact, the development of such multitarget drugs has already been suggested, and research in this field has started (Carroll 2005; Ramsay et al. 2018). Studies on new potential anticancer drugs are examples of the use of such strategies. For instance, multikinase inhibitors have been intensively studied and include pyrido(2,3-d)pyrimidin-7-one derivatives, which act as dual inhibitors of cyclin-dependent kinases 4 and 6. Some of them can be used in breast cancer therapy (Asghar et al. 2015). Another multikinase inhibitor is midostaurin (4’-N-benzoylstaurosporine), which negatively regulates the activities of several enzymes (PKCa, VEGFR2, KIT, PDGFR, and FLT3). It is used in the treatment of patients with acute myeloid leukemia and potentially might be employed in therapies for related diseases (Levis 2017). On the other hand, one might predict that such drugs might be effective in the treatment of a particular single disease or a group of similar diseases (e.g., different kinds of related cancers) rather than a large group of different diseases. Nevertheless, another example is the employment of anti-inflammatory molecules that might be used to alleviate symptoms of many diseases, since inflammatory processes have been recognized as those either causing or worsening many different disorders. For instance, infliximab (a monoclonal antibody) is a therapeutic molecule that binds and inactivates TNFα, thus reducing inflammation, and it was found to be effective in improving the conditions of patients suffering from various diseases, including Crohn’s disease, ankylosing spondylitis, rheumatoid arthritis, and psoriatic arthritis (Melsheimer et al. 2019). Regardless, these diseases are not rare, as are viral diseases, for which different kinds of novel multitarget drugs and nucleoside analogs, have been developed recently (Pastuch-Gawołek et al. 2019).

Clearly, if we want to find one drug for many rare diseases, it is necessary to find a biological process that might result in eliminating the cause of various disorders (either only rare or both rare and common diseases). Let us consider diseases that are caused by the accumulation of excess abnormal molecules. There are many such diseases, including lysosomal storage diseases, in which accumulation of various incompletely degraded molecules causes cellular dysfunctions, disorders caused by the aggregation of abnormal forms of various proteins, including Huntington’s disease or amyotrophic lateral sclerosis, and Alzheimer’s disease (definitely not a rare disease), in which the accumulation of β-amyloid and hyperphosphorylated tau proteins occurs. It was proposed that the stimulation of the autophagy process, leading to the degradation of abnormal macromolecules and even destruction of whole abnormal cellular organelles, might provide a method for the elimination of causative agents of numerous diseases (Pierzynowska et al. 2018a). This could potentially solve the problem of the economical trap, as the number of patients would be high, because the potential drug could be used for the treatment of both rare and common diseases. However, the question remains how to find an effective autophagy stimulator that is safe enough to be used as a long-term treatment (patients suffering from genetic diseases and neurodegenerative disorders need life-long therapies) and can reach the brain. The vast majority of already known autophagy stimulators are effective in vitro, and some of them can even be used for short-term treatment; however, the adverse effects that they cause preclude their administration to patients for years (Pierzynowska et al. 2018a). Nevertheless, recently published reports indicated that compounds having all desired features may indeed exist. A small molecule, genistein (5,7-dihydroxy-3-(4-hydroxyphenyl)chromen-4-one), was found to be effective in stimulating autophagy in different experimental systems (Hasima and Ozpolat 2014; Pierzynowska et al. 2018b; Pierzynowska et al. 2019). This compound was effective in eliminating causative agents in cellular and animal models of different diseases, including mucopolysaccharidoses (a group of lysosomal storage diseases), Huntington’s disease, and Alzheimer’s disease, and its administration resulted in the complete correction of animal behavior (Malinowska et al. 2010; Pierzynowska et al. 2018b; Pierzynowska et al. 2019). However, despite surprisingly high efficacy of genistein in tests on animal models of these diseases, it is not possible to predict if this molecule is also efficient in therapies of humans. Overall, this is an example of a single compound that can be used to treat various conditions in vivo and whose molecular mechanism of action provides an explanation of how cellular process modulation may abolish the major causes of different diseases.

### Concluding remarks

In summary, the above-discussed results of studies on multitarget drugs and modulators of processes that might be common therapeutic targets in various diseases indicate that it is possible to find molecules that fulfill the requirement of an effective drug that may be used to resolve the economical trap of rare diseases. Therefore, further preclinical and clinical studies on these compounds and different molecules that can safely modulate specific processes that might result in the amelioration of conditions of patients with many different rare diseases are required to test the hypothesis presented in this article. Such studies are neither easy nor cheap, but one may consider conducting them to assess if the possibility of unlocking the economical trap of rare diseases is real.

## Data Availability

Not applicable.
